# The pattern of educator voice in clinical counseling in an educational hospital in Shiraz, Iran: a conversation analysis

**Published:** 2017-10-18

**Authors:** Ahmad Kalateh Sadati, Kamran Bagheri Lankarani

**Affiliations:** 1 *Assistant Professor, Department of Social Sciences, Yazd University, Yazd, Iran.*; 2 *Professor, Health Policy Research Center, Institute of Health, Shiraz University of Medical Sciences, Shiraz, Iran.*

**Keywords:** *Clinical consultation*, *Doctor-patient interaction*, *Educator voice*, *Conversation analysis*

## Abstract

Doctor-patient interaction (DPI) includes different voices, of which the educator voice is of considerable importance. Physicians employ this voice to educate patients and their caregivers by providing them with information in order to change the patients’ behavior and improve their health status. The subject has not yet been fully understood, and therefore the present study was conducted to explore the pattern of educator voice. For this purpose, conversation analysis (CA) of 33 recorded clinical consultations was performed in outpatient educational clinics in Shiraz, Iran between April 2014 and September 2014. In this qualitative study, all utterances, repetitions, lexical forms, chuckles and speech particles were considered and interpreted as social actions. Interpretations were based on inductive data-driven analysis with the aim to find recurring patterns of educator voice. The results showed educator voice to have two general features: descriptive and prescriptive. However, the pattern of educator voice comprised characteristics such as superficiality, marginalization of patients, one-dimensional approach, ignoring a healthy lifestyle, and robotic nature. The findings of this study clearly demonstrated a deficiency in the educator voice and inadequacy in patient-centered dialogue. In this setting, the educator voice was related to a distortion of DPI through the physicians’ dominance, leading them to ignore their professional obligation to educate patients. Therefore, policies in this regard should take more account of enriching the educator voice through training medical students and faculty members in communication skills.

## Introduction

The educated patient is a person who is trained in different aspects of therapeutic measures and health, and is informed about promotion of clinical health by a doctor (1). Education improves patients’ satisfaction with the doctor-patient interaction (DPI), the quality of care, and the reduced health care costs resulting from patients’ enhanced knowledge of health issues (2). Redman believes that the main goal of patient education should be to direct the application of physician's prescribed actions (3). The clinical setting is partly responsible for educating patients, since the goals of clinical teaching and learning are based on patients’ assessment, evaluation, diagnosis, prognosis, and individuals’ needs in relation to intervention (4). The desirable interaction between doctor and patient makes it possible to design better educational strategies and training. This empowers physicians to interact with their patients using greater technical and relational efficiency (5). Thus, DPI is crucial in medical research and sociology of medicine, and plays an important role in the education of patient (6 - 8).

Based on sociological theories, there are different voices in every DPI (7 - 9) that work in conjunction with each consultation. An important voice is the educator voice (EV) that conveys various kinds of medical information to patients in the hope of getting a better understanding of their health problem. The principal function of EV is to bring about patients’ compliance and consequently facilitate communication of medical information (7). Promoting patients’ understanding to follow the appropriate treatment is the primary purpose of EV as a component of DPI that facilitates health awareness and education of patients. 

Patient education is the most important issue of the health system in developed countries. Visser et al. showed that patient education represents a general feature of healthcare in the UK, Belgium, Germany, and Netherlands (10). In addition, it is defined as a rightful (11), and an ethical (12) issue. In Iran, however, although updated texts underlining the importance of patient education are included in medical education syllabus, there is as yet no information about the pattern and quality of patient education in relation to DPI, which is shown by recent studies to be distorted (6, 13, 14). In this form of interaction, the consultation is conducted within a few minutes, followed by the patient leaving the physician’s office with only a written prescription, and there is usually little or no communication between the doctor and patient (13). In clinical settings, patient education is realized through DPI, which adequately informs the patient about his/her health. Therefore, the aim of the present study was to explore the pattern of EV in clinical settings and address the way the doctors educate patients during consultation and also the general characteristics of EV.

## Method

This was a qualitative study based on conversation analysis (CA) derived from ethno-methodology. CA is a traditional method in sociology introduced by Goffman (15) and published as early as 1974 by Schegloff and Sacks (16). In sociology it has been defined as the science of natural observation (17-19) aiming to study the subjects by detailed inspection of tape recordings and their transcriptions (16). CA identifies the patterns of behavior as well as interactional strategies, and explores the association between certain interaction styles and outcomes (20). 

The data were collected between April 2014 and September 2014 in one outpatient educational clinics, affiliated with Shiraz University of Medical Sciences (SUMS). The hospital included endocrinology, anesthesiology, general surgery, gynecology, neurology, urology, dermatology, and internal medicine clinics. All specialties in this clinic except for neurology and gynecology were included. The number of samples was determined based on saturation. Purposeful sampling was carried out with variations in some parts. Inclusion criteria for selecting specialists comprised having more than 3 years of teaching and clinical experience, being a member of the university at the time of the study, and not being retired or employed at other medical training facilities. Having briefed the specialists about the objective of the present research, 9 physicians with 7 different specialties agreed to participate in the study. At this stage, the surgeons expressed their unwillingness to cooperate and were therefore excluded. 

The researcher obtained consent from 50 patients to make the recordings. The recorded data were then transcribed by an independent researcher who was aware of the aim of the study, and was also familiar with CA and the method of transcribing data in conversation form. The data transcription included all repetitions, elisions of lexical forms, use of temporal regulators, chuckles, utterances such as “ahem, yeah, you know, right”, and speech particles like “uh, oh”, etc., specifically short syllabic devices. Although transcription is a selective process (16), the conversations were fully transcribed. Overall, 50 conversations were transcribed of which 33 were analyzed based on saturation criteria (21, 22, 23). Inclusion criteria for selecting conversations comprised lasting more than three minutes and involving all specialties, with the most frequent conversations receiving priority. 

After transcription, an inductive data-driven analysis was conducted to find recurring patterns of interaction. Analysts developed conversation-related rules or models to explain the frequencies of the patterns. On the other hand, the conversations were evaluated as social actions, since human social actions are thoroughly structured and organized by conversation. Therefore, transcribed data were analyzed considering meaningful episodes of talk and their interpretations, a step that was repeated in subsequent stages (16). Generally, conversation analysis involved all utterances and many aspects of non-verbal behavior in relation to social actions of various kinds, and those features that are generally linked with broader activities arising during consultation (20). However, in this study, video recordings were not used, and only the verbal data were analyzed. Therefore, researchers emphasized the pattern of EV as a social action that occurred during the verbal interaction of both parties. 

The validity of the study was confirmed in three ways. The first step was member check, reflecting the researcher’s feedback on interpretations by some of the participants (22). For this purpose, researchers presented the findings to participating physicians to obtain their validation of the extracted rules. The patients, on the other hand, could not be accessed, and therefore the results were verbally obtained from 17 patients who referred to the clinics and confirmed the findings. Second, trustworthiness was observed during the study by ensuring subjectivity and reflexivity, adequacy of data, and efficiency of the interpretation strategies (22). In this context, we shared our findings with the research team concerning the rules extracted from the data, and fully agreed with them on inter-subjectivity characteristics. Third, the credibility was ensured by member check and peer debriefing on the foregoing items, and involving original informants or others with similar responsibilities (23). We then checked the initial findings with the aforementioned participants and reached a collective agreement in each step. In addition, reflexivity was observed by emphasizing production of knowledge and minimizing prejudgments by researchers, a process carried out through continuous monitoring the results of the study.

With regard to ethical considerations, alongside obtaining the consent of participants for taking part in the study, attempts were made to maintain anonymity of the participants and confidentiality in all stages, from data collection to the final research report. Furthermore, the study was approved by the Ethics Committee of Shiraz University, Iran in 2014. It should be noted that the transcriber pattern in this research is based on the models proposed by Atkinson and Heritage (24) and Heritage (25). 

## Results

Results showed that 7 specialties participated in this study. The characteristics of the participants are shown in Table 1. 

**Table 1:** Characteristics of the research participants

**Type of Specialty**	**Number(s) of Participants**	**Gender**	**Practical Experience (Year)**
Internist	1	Male	4
Infectious disease specialist	1	Male	9
Endocrinologist	1	Female	4
Urologists	3	Male	12 (one of the participants) 5 (2 of the participants)
Dermatologist	1	Male	15
Anesthesiologists	1	Female	17
Rehabilitation specialist	1	Male	11

With regard to the EV in clinical conversations, the obtained results were expected to address the general quality of EV and its characteristics during consultations by answering the following questions:

**1. How do doctors educate patients during consultation sessions?**

Based on the conversations, doctors educated their patients by descriptive and prescriptive methods. Descriptive voice is related to informing patients about the existing situation, whereas prescriptive approach refers to patients’ duties and obligations. 

*Descriptive Voice* *:* In this voice, a doctor tells the patient about the etiologies of illnesses, duration of treatment, stages involved in the recovery process, and the graphs concerning paraclinical results. As an instance, see consultation 14 below, offered to a patient with H. pylori infection. Note that in all the following samples, *D* will be used to represent the doctor and *P* the patient:

*D: Yeah, duodenal ulcer is not usually cancerous.*

*P: OK…*

*D: Typically it recovers faster.*

*P: Oh… really?*

*D: Yes, usually. You don’t have to do another endoscopy if symptoms of infection with H. pylori and peptic ulcer disappear.*

*P: Thanks!*

As shown in these interactions, the physician uses paraclinical data to convince the patient that his condition has improved, and also ensures him that there is no need for a second endoscopy. In addition, sometimes physicians inform their patients about the course of the illness and possible treatments. For example, in consultation 22 involving renal colic, the physician informs the patient in the following way:

*D: Your urine analysis report shows that there is some blood in your urine, but that’s OK… it seems that your kidneys are clean.*

*P: No, this isn’t the last test.*

*D: This one is the latest.*

*P: Yeah, this is it.*

*D: Have you seen a stone pass through your urine lately?*

*P: No.*

*D: This is also good news, probably it has passed.*

In this situation, the physician refers to the urine analysis in order to inform the patient that the renal stone has probably passed. Thus, in this short conversation, the physician explains the process of the illness to the patient. Sometime the physician talks about the prognosis of illness in descriptive voice. For example, a physician in consultation 6 states: 

*D: Your kidneys are better, your anemia is OK too, but your blood sugar… is not under control.*

In this statement, the physician seeks to explain the overall condition to the patient. Additionally, the physician informs the patient that his/her blood sugar needs to be checked regularly. 

*Prescriptive* *Voice:* Here, the doctor includes various issues concerning the patient’s duties such as next referral, paraclinical tests, graphs, medication prescription and self-care. This is the main part of EV that deals with promotion of health by prescribing medication as well as reminding the patients to have healthy behavior. However, the dominant issues to be considered comprise medication and further tests. For example, in consultation 9 concerning a woman with acne, the physician’s instructions and prescription were as follows:

*D: These are the tests that you should do on the third and fifth day of your menstruation period. I have prescribed some medications. Take a capsule with your lunch every other day. If you get parched lips, use a chap stick, and see me in one month with your tests results.*

Consultation 16, offered to a woman with advanced breast cancer, was dominated by paraclinical data and the following prescription:

*D: This is a sleeping pill that you should take before going to bed, but if you take it at dinnertime, you should go to bed immediately. I have also ordered a chest X-ray. Remember to hold your breath while it is being taken. Please see me in three weeks, and bring your X-ray results.*

As can be seen above, the consultation included prescribing medication, taking an X-ray, and setting a future appointment. 

**2. What are the general characteristics of EV?**

Considering the aforementioned characteristics and the critical analysis of EV and the transcripts, several flaws were detected in the consultations, which had the following characteristics. 

*Superficiality*

EV can be superficial, because it may not focus on important issues. Nevertheless, such issues are not difficult to address and can be resolved by evaluating the consultation sessions that contained nothing concrete. For example, diabetes is a chronic disease that needs special education. In consultation 6, an illiterate elderly woman referred to an endocrinologist who discussed many issues such as the patient’s history, medication and the range of blood test without offering any training or education. The patient only learnt from her physician that she had high blood sugar denoted by an FBS of 303. The physician did not pay any attention to other aspects of the educational needs of an illiterate, elderly woman such as diet or exercise. At the end of this consultation the physician changed the patient’s medication and asked for a retest of blood sugar.

*D: Um… the tests are not good. Your blood sugar is high. Now, I’ll change your pill. Take this new pill.*

*P: Thank you.*

*D: Goodbye for now.*

*Marginalization of Patients*

Consultations showed that EV is merely based on the physician’s domination, where the doctor makes a decision about a patient’s education without his/her involvement. For example, in consultation 33, regarding a woman with numbness in her shoulder, the patient had previously been prescribed to do the nerve conduction velocity (NCV) test. Based on her test results, her physician recommended surgical operation, but she refused to comply due to her fear of having no one to look after her and uncertainty about the diagnosis. She then asked her doctor for an alternative procedure. Since the physician’s opinion was based on the NCV results, he tried to ensure her that what he recommended was the best choice, and although he was aware of the patient’s doubts, he insisted that she should comply. In this connection, the underlying verbal exchanges between the doctor and the patient are worthy of consideration:

*D: Why don’t you want to undergo the operation?*

*P: Well, I think I may not (really) need an operation, am I (not) right?*

*D: According to this nerve strip (NCV),* *you should do it!*

*P: Well, isn’t there anything else to do?*

*D: You see, nothing is certain. What I mean is that this nerve strip (NCV) says you need to undergo operation, and scientifically speaking* ***,*** *I am sure we will be able to achieve good results.*

*P: Yeah.*

*D: A patient like you should undergo this operation. If you disagree or resist, you won’t get the desired outcome.*

*P: But I won’t do it.*

*D:* *Listen! Resistance means you are avoiding the operation, which should be performed under any circumstances.*

In this consultation, due to the dominance of the physician’s voice, the EV is asymmetric. In other words, the descriptive voice is based on the nerve strip and the physician’s prescription and dominance, as well as his insistence on a surgical option without paying any attention to the patient’s concerns. In this situation, the slightest doubt in the patient’s mind can lead to the failure of EV. The reality is that the patient is deeply marginalized, and her wisdom and knowledge is being completely ignored by the physician. 

Overall, when no attention is paid to the patient’s standpoints, the consultation will likely fail and creates psychological pressure on the patient. Of the 33 consultations in this study, there was only one case in which the physician (an urologist) provided adequate explanations to reduce psychological stress on the patient’s family member. In this case (consultation 23), the patient’s son had tried to conceal his mother’s illness, which was a kidney tumor. 

*One-Dimensional Approach*

Another critical characteristic of all consultations was that they were one-dimensional, in that the physician’s approach was merely based on his/her specialty domain. For example, a surgeon only paid attention to the surgical procedure, an attitude predominant in several fields of medicine. In this case, other disciplines are generally ignored, unless the patient complains. 

For example, in consultation 6 that was offered to a diabetic patient, the physician did not pay any attention to the effects of high blood sugar on other organs such as the heart, eyes and the nervous system. Another example pertains to consultation 18 involving a patient with spinal cord injury who was afflicted with a testicular and urinal infection. In this case, the patient’s son talked about his father’s digestive problem due to antibiotic consumption, but the physician ignored his concerns and proceeded to conclude the session. The conversation between the patient’s son (PS) and the doctor is presented below: 

*PS: Thank you. Aren’t you going to prescribe any drugs?*

*D: No, there is no need for drugs at the moment, just use the previous ones.*

*PS: Well, those cause diarrhea!*

*D: If that is the case, clindamycin should not be taken.*

*PS: Isn’t there any alternative medicine; something that alleviates his stomach discomfort and painful bowel movement? These conditions are annoying to others.*

*D: OK, have a good day!*

*Ignoring a Healthy Lifestyle*

A healthy lifestyle is one of the most important dimensions of the educator voice that has to be considered by a physician. This includes proper information on healthy behavior, diet, exercise, and hygiene. However, an evaluation of the consultations revealed that a healthy lifestyle was generally ignored and the doctor’s duty was simply limited to prescribing medication, although in some sporadic cases this issue was touched upon by the physician. For example, in consultation 4 involving a patient with anal fissure, the physician indicated that being overweight is the main cause of the problem, but her advice was limited to the following statements:

*D: How much do you weigh?*

*P: Umm… 73 Kg.*

*D: You are slightly overweight. Yeah, you’re too young to be this weight.*

*Patient’s Mother: He doesn’t eat much but he gains weight.*

*D: He does not have enough exercise.*

In this conversation, the physician’s statements focused on her observation and did not provide any solution to the problem in order to promote a healthy lifestyle. In other consultations, such as those with diabetic, elderly and obese patients, recommendations with regard to a healthy lifestyle were totally overlooked. 

*The Robotic Nature of Consultations*

This theme means that essentially all consultations were based on paraclinical observations and only occasionally did they include physical examinations or active conversations. In this situation, patients felt that the consultations were meaningless. A robotic consultation frequently involves routine exchanges such as “Let me see your echo test results”; “Your tests are incomplete according to the ultrasound”; “Have you brought your previous tests?”; “Your previous test results were better than the present report”; “You should do another test and come back in one month”; “I will prescribe a re-test”; “Repeat the test”; “Just do these tests”; “For the time being, your treatment depends on the results of these tests”; “Bring the results to me later”; “An endoscopy re-test might be needed”; “Let me check your blood sugar too”; “I also recommend a mammography”; “You should get scanned in two months” and so on. 

These types of clinical approaches can be evaluated from several aspects, but when viewed as EV, they appear robotic. In this case, the physician merely relies on paraclinical data and ignores the other features of a consultation. Therefore, the question is, what happens if the paraclinical data is not accurate or is misleading, a situation that does not clearly define the impact of EV.

## Discussion

This study shows that the EV employed by physicians is expressed in either descriptive or prescriptive manners. In this context, those characteristics of EV that are related to the nature of the DPI include superficiality, marginalization of patients, one-dimensional approach, ignoring a healthy lifestyle, and the robotic nature of consultations. Generally, a weakness or lack of educational aspects was detected in the doctor-patient communication within the context of this research. As shown by several studies, DPI is distorted and asymmetrical (6, 13, 14) due to the paternalistic quality of the doctor-patient relationship (6). In this regard, another study showed that patients experienced interactions with unexpected, unprofessional, instrumental, and non-cooperative features (26). These findings are totally in line with our study, in that they confirm the inappropriate patterns of EV and DPI, and an irrational relationship that disregards the patients’ educational needs.

For example, the physician visits the patient as a faceless and inattentive robot with weak interaction skills (14), and this is a situation that reflects an asymmetrical power relationship in DPI (6). Therefore, under these circumstances, the discourse prevailing a doctor’s visit is expressed as his or her active dominance resulting in marginalization of patients, one-dimensional approach, and the robotic nature of DPI. 

Despite the doctor’s dominance, the modern approach underlines patients’ concern for acquiring an understanding of their disease (27). In this approach, a physician is compelled to consider the patient’s worries and apprehensions, a situation that involves the patient in the treatment process. Accordingly, EV can be improved by minimizing patient marginalization and the robotic traits of the consultation, and listening to the patient’s viewpoints attentively instead. 

Cordella critically reviewed the nature of EV in clinical counseling and discussed the deficiencies in 7 consultation sessions, posing a number of main questions including: “Are we to understand the absence of the EV in those consultations in which seven patients were deprived of the opportunity to acquire a better understanding of their health condition?”; “Will this limit their chances of adequately looking after themselves?”; “Is the absence of patient education in almost one-third of the consultations contradictory to the basic teaching principles of the institution where the study was conducted?”; and “If we accept that the silencing of EV may be a problem, what could be its underlying cause, and how should it be interpreted?” (7). our investigation confirms Cordella’s findings, while regarding her last question, the cause can be explained as follows: 

It is difficult to determine whether or not we are faced with the silent EV. Even though EV is silent overall, some physicians tried to respond to the patients’ questions as thoroughly as possible. Generally speaking, it seems that an obvious deficiency in EV lowers the patient’s confidence. However, if physicians actively assist patients in the information gathering process, it will result in an improved relationship, which is a potential gain for all collaborative parties (28). Patients’ mistrust in their doctor has been reported by several studies (14, 27, 29). In addition, research has shown that the recent Health Sector Evolution Plan in Iran has intensified a distorted DPI (30). In this situation, it is clear that the silent EV generally leads to patients’ reduced confidence in DPI and their non-compliance to prescriptive voice. 

According to the findings of the present study, which to our knowledge is the first on this highly important topic, the existing EV is unacceptable. The obtained results can provide a guideline for future studies comprising qualitative or quantitative approaches. It also shows that distorted DPI is related to a deficiency in EV, an area to be evaluated in future quantitative studies. Finally, because of the significance of EV, this research recommends that health policy makers pay more attention to providing the means for better patient education throughout the country. One limitation of this study was the way in which the conversations were recorded. In fact, recording affects doctors’ manners of consultation and dialogue. However, the researchers did not find any other alternative for data collection. This is an important and basic restriction of qualitative studies since the participants are aware of the recording and data collection, even though in this case the researcher did not interfere with the natural course of the dialogs. Thus, although recording the data considerably reduced the bias, it could have affected the quality of consultation, and the resulting pattern may not show some aspects of the existing behavioral patterns in DPI. Another limitation of the present research was that the type of disease, the patient’s gender and even the way the patient interacts with the physician affect the DPI, but such factors tend to be ignored in qualitative studies due to the very nature of this type of research. 

## Conclusion

EV is an important part of any clinical consultation. In this context, some issues that were noted with EV included superficiality, marginalization of patients, one-dimensional approach, ignoring a healthy lifestyle, and the robotic nature of consultations. These characteristics created a substantial problem in DPI, one important function of which was to promote the patient’s health-related awareness. The silent EV does not occur frequently, but the findings of this study showed an obvious deficiency in the current state of EV. An important point is that those in charge of the Iranian health system should be aware of the weaknesses in clinical consultation and solve the problems scientifically. In this regard, health policy makers should pay more attention to effective EV with regard to training medical students and faculty members in communication skills. Since these are not included in the current curriculum of medical education, it is recommended that the authorities solve the problem by holding related courses for all medical groups in the short term, and by defining such courses in the long term.
